# Genetic susceptibility associated with hospitalization due to respiratory syncytial virus in a group of Taiwanese children: a preliminary study

**DOI:** 10.3389/fped.2025.1473448

**Published:** 2025-08-12

**Authors:** Yi-An Lu, Chi-Jen Chen, Tzu-Pin Lu, Jin-Yuan Wang, Daniel Tsung-Ning Huang

**Affiliations:** ^1^ Division of Pediatric Infectious Diseases, Department of Pediatrics, Mackay Memorial Hospital, Taipei, Taiwan; ^2^ Institute of Epidemiology and Preventive Medicine, Department of Public Health, College of Public Health, National Taiwan University, Taipei, Taiwan; ^3^ Institute of Health Data Analytics and Statistics, Department of Public Health, College of Public Health, National Taiwan University, Taipei, Taiwan; ^4^ Department of Medicine, Mackay Medical College, New Taipei City, Taiwan

**Keywords:** genetic polymorphism, respiratory syncytial virus, Taiwanese pediatric population, hospitalization, risk factor

## Abstract

**Background:**

Respiratory syncytial virus (RSV) is a leading cause of lower respiratory tract infections in young children worldwide. While several risk factors for severe RSV illness are known, the role of host genetic susceptibility remains underexplored, particularly in East Asian populations. Objective: This preliminary study aimed to identify genetic variants associated with RSV-related hospitalization in the Taiwanese pediatric population using a genome-wide association approach.

**Methods:**

A total of 260 children aged ≥6 months were recruited from Mackay Memorial Hospital and the corresponding author's social media page between November 2020 and March 2022. Genotyping was performed using the Axiom Genome-Wide TPM 2.0 array, followed by imputation and quality control. Genome-wide association analyses were conducted under additive, dominant, and recessive models, adjusting for population stratification.

**Results:**

Eight single nucleotide polymorphisms (SNPs) were significantly associated with RSV hospitalization risk (rs183825, rs141541148, rs7296788, rs16862251, rs1525107, rs2105758, rs622946, and rs12857032). Notably, rs141541148 (OR = 9.14) and rs1361088 (OR = 8.58 in boys) were associated with substantially increased risk. Conversely, rs16862251 was linked to a reduced risk (OR = 0.19), suggesting a protective role possibly mediated through T-cell receptor signaling.

**Conclusion:**

Our findings identify several gene loci associated with higher rates of hospital admissions due to RSV in children of ≥6 months of age. By studying the genetic variations that may predispose people to RSV infection, it may be possible to gain a better understanding of risk factors and prioritize vaccination for specific populations.

## Introduction

In recent decades, there have been significant advances in understanding the genes that impact susceptibility to and severity of pathogens such as viruses, bacteria, and parasites. Through advancing our understanding of pathogen–host interactions, these studies can have important implications for disease prevention and treatment, as well as for the development of personalized vaccines. This is important, especially for children, as they may benefit greatly from this research.

Respiratory syncytial virus (RSV) is the most prevalent lower respiratory virus in young children; it is also an important etiological cause of respiratory illnesses (1, 2). There were an estimated 3.6 million hospital admissions due to RSV-associated acute lower respiratory infection globally in children aged 0–60 months (2). By 2 years of age, nearly all children have been infected at least once, with 1%–2% requiring hospitalization (3, 4). RSV represents significant public health burdens in Taiwan, particularly among children and the elderly. A study analyzing surveillance data from 2010 to 2020 found that RSV infections increased significantly in Taiwan, becoming the most common respiratory viral pathogen in 2020 (5). This shift highlights RSV's growing role in respiratory illnesses within the country.

Several predisposing factors may contribute to the severity of RSV disease. However, severe RSV-related illness has been diagnosed in individuals not considered to be high-risk candidates for severe RSV disease. By studying the host's genetic factors, particularly the genes associated with the innate immune response, it may be possible to gain a better understanding of risk factors and consequently prioritize vaccination for specific populations (6). Several genome-wide association studies (GWAS) have identified variants in genes related to immune response. Notably, most of these findings stem from studies conducted in Western or multiethnic populations, with limited data from East Asian or Taiwanese cohorts, highlighting a gap that this study seeks to address.

Thus, to determine common genetic variants for the risks of RSV hospitalization in Taiwanese Han children, the current study applied a genome-wide array approach. We aimed to identify the gene loci responsible for higher hospital admission rates due to RSV in children of ≥6 months of age.

## Methods

### Study population

Mackay Memorial Hospital and the corresponding author's social media page served as sources to recruit participants between November 2020 and March 2022. The ethical approval was obtained from Mackay Memorial Hospital Institutional Review Board (approval no: 20MMHIS181e). Data collection involved questionnaires and oral mucosal specimen. Participants were recruited via a post on the corresponding author's social media page, which has over 800,000 followers, with approximately 95% being native Taiwanese parents, inviting parents of potentially eligible participants to complete an online questionnaire. Upon receipt of the questionnaire, the research team reviewed the responses to confirm eligibility and willingness to participate. Eligible participants and their legal guardians received an informed consent form, which was completed and returned to the research team. Once the consent form was verified, the DNA collection kit was mailed by the collaborating laboratory to the participants' homes. Parents were instructed to collect oral mucosal samples from the children and return the specimens directly to the laboratory for analysis.

The questionnaire investigated gender, age, residence location, prior infection history, vaccination history, and disease status. To enhance data reliability, the questionnaire included logical consistency checks to identify contradictory answers. Diagnoses of RSV were determined based on these structured reports, reflecting typical clinical practice involving PCR or rapid testing. The case group included children who had been admitted for RSV at any time from 6 months of age up to the time of recruitment. Controls were matched by sex and by age (within ±3 months) at the time of recruitment.

**Figure 1:**
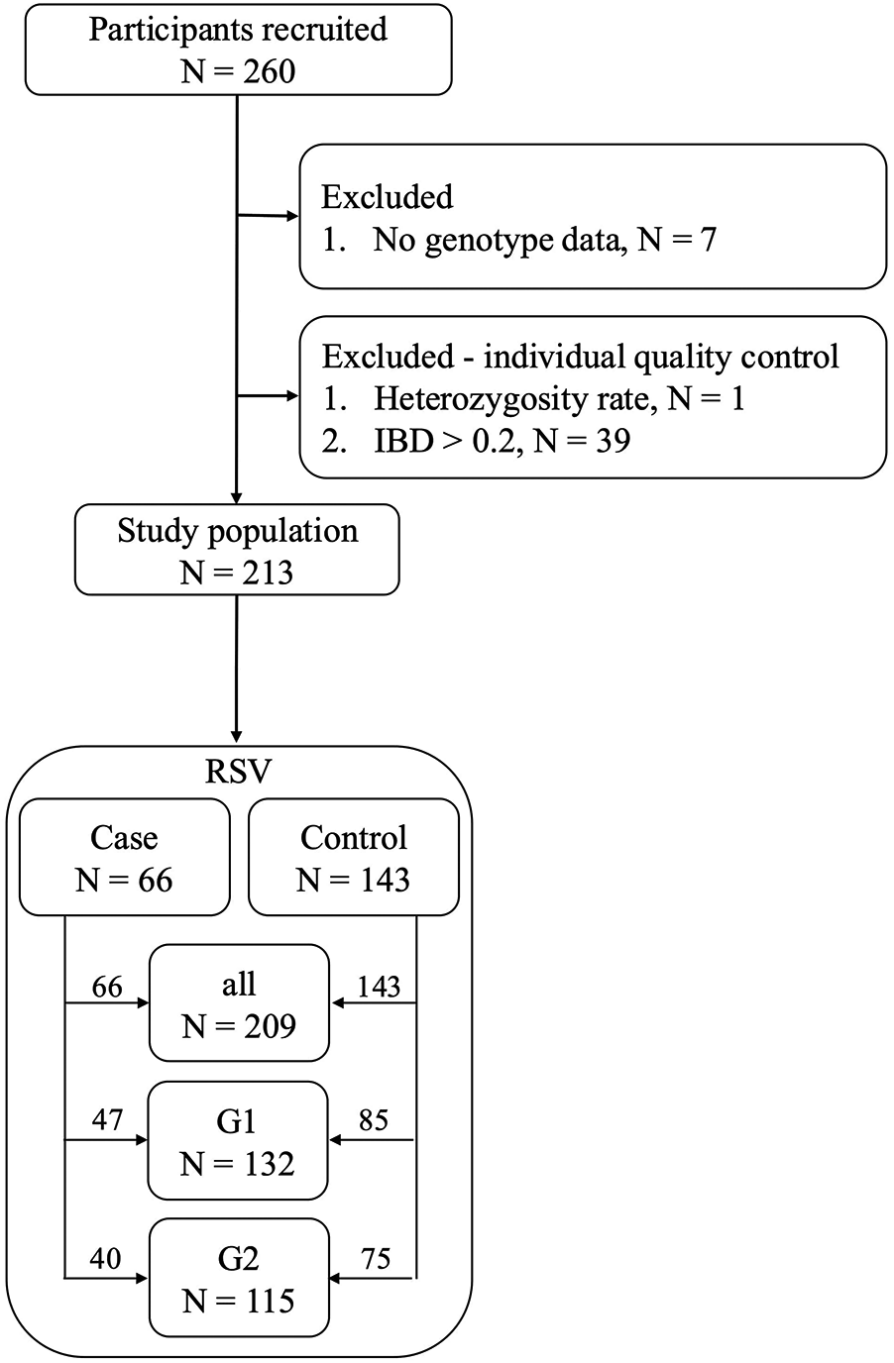
Flow chart. IBD, identical-by-descent; RSV, respiratory syncytial virus.

Before determining the final study population, we first excluded individuals without genotype data. During the individual quality control process, we also excluded those with excessively high or low heterozygosity rates, as this could indicate contamination during the sequencing experiment or potential inbreeding among the samples. Additionally, individuals with an identical-by-descent (IBD) >0.2, indicating a relationship within three degrees of kinship, were excluded (Figure 1). The population was further divided into three groups based on residential areas and sex. Specifically, cases in Group 1 (RSV G1) included only boys who had been hospitalized with RSV infection, along with male controls who had not, due to sex-related differences in risk (7). Group 2 (RSV G2) included only individuals residing in the Taipei and New Taipei City area, due to rural–urban healthcare disparities, with cases who had been hospitalized for RSV and controls who had not. Group 3 (RSV all) combined RSV G1 and RSV G2 groups, excluding duplicates. Detailed definitions of all groups are summarized in Supplementary Table S1.

### DNA extraction and genome-wide association study array

DNA was extracted from the oral mucosa using the MagMAX™ DNA Multi-Sample Ultra 2.0 Kit in accordance with the operation manual (MAN0017326-REV.C.0). The DNA samples were sent to the National Center for Genome Medicine for analysis using the Axiom Genome-Wide TPM2.0 Array (Thermo Fisher Scientific). The genotypes were analyzed, genotype raw data were obtained, and test reports were generated.

### Imputation and quality control

In this study, genotype imputation was performed to increase the number of genetic variations by inferring missing or unknown genotypes through known genotypes from multiple individuals. We used the online Michigan Imputation Server (Minimac4) with the 1000 Genome Phase 3 (GRCh38) reference panel. To ensure the quality of the genotypes, we excluded those with an *r*^2^ value <0.3. Consequently, the number of genetic variations increased from 699,246 to 13,279,316.

To ensure the accuracy, reliability, and consistency of the genotype data, we performed quality control, which mainly included individual and SNP quality control. We excluded genetic variations that did not meet the standard criteria of this study. SNP quality control was performed using the following three criteria: SNPs with a call rate of <99%, a Hardy–Weinberg of equilibrium *p* < 10^−6^, and a minor allele frequency of <1%. The individual quality control used the following three criteria: individual call rate of <99%, extremely large or small heterozygosity rate, and related individuals with identity-by-descent status of >0.2.

### Genome-wide association study

After the quality control of each phenotype, we conducted a genome-wide association study. In the genome-wide association analysis, we used different genotype models to explore the results. We used additive, dominant, and recessive models to describe the relationship between alleles and the risk of RSV. Then, we used logistic regression to calculate allelic effects on RSV. To mitigate the effects of population stratification, we performed principal component analysis and adjusted for principal components 1–3. The threshold for genome-wide significance was set as *p* < 10^−5^. The analysis was conducted using PLINK v2.0 (https://www.cog-genomics.org/plink2) and Python 3.7.

## Results

### Study population and variant quality control

Following quality control, the study population decreased from 260 to 213 participants, and the number of genetic variations was reduced from 13,279,316 to 7,589,740. The demographic characteristics, presented in Table 1, participants had a mean age at recruitment of 6–7 years, with a higher proportion of boys compared to girls (63.16% vs. 36.84%).

**Table 1:** Demographic characteristics of children hospitalized with RSV.

Characteristic	RSV All	RSV G1	RSV G2
Age (years)			
Mean	7.08	6.86	7.28
S.D.	5.25	4.17	6.26
Gender			
Boy	132	132	79
%	63.16	100.00	68.70
Girl	77	-	36
%	36.84	-	31.30

RSV G1, respiratory syncytial virus group 1; RSV G2, respiratory syncytial virus group 2.

### Genome-wide associations in the overall RSV cohort

The genome-wide association study (GWAS) revealed significant associations in different genetic models for all RSV groups in Table 2. In the additive model, three SNPs [rs183825: OR = 6.62, *p* = 4.13 × 10^−6^, 95% confidence interval (CI) = 2.96–14.79; rs141541148: OR = 9.14, *p* = 2.26 × 10^−6^, 95% CI = 3.6–522.86; rs7296788: OR = 0.28, *p* = 1.70 × 10^−6^, 95% CI = 0.16–0.47] were significant. These SNPs were located in intergenic regions between the von Hippel–Lindau tumor suppressor and IRAK2 (interleukin 1 receptor associated kinase 2) on chromosome 3p25.3, SELENOT (selenoprotein T) and ERICH6 (glutamate rich 6) on chromosome 3q25.1, and in the coding region of KSR2 (kinase suppressor of ras 2) on chromosome 12q24.23.

**Table 2:** Top SNPs identified from genome-wide association analysis of RSV hospitalization in the combined group.

SNP ID	rs ID	Location	Gene	Genotype	Patient group (*n* = 66)	Control group (*n* = 143)	Model	*p*-value	OR (95% CI)
3:10163219:C:T	rs183825	3p25.3	Intergenic	TT	1 (1.52%)	0 (0.00%)	Additive	4.13 × 10^−6^	6.62 (2.96–14.79)
				TC	22 (33.33%)	12 (8.39%)	Dominant	4.15 × 10^−6^	6.77 (3.00–15.27)
				CC	43 (65.15%)	131 (91.61%)			
3:149790142:G:A	rs16862251	3q25.1	ANKUB1	AA	3 (4.55%)	14 (9.79%)	Dominant	2.02 × 10^−6^	0.19 (0.09–0.37)
				AG	12 (18.18%)	69 (48.25%)			
				GG	51 (77.27%)	60 (41.96%)			
3:150655101:T:C	rs141541148	3q25.1	Intergenic	CC	0 (0.00%)	0 (0.00%)	Additive	2.26 × 10^−6^	9.14 (3.65–22.86)
				CT	22 (33.33%)	8 (5.59%)	Dominant	2.26 × 10^−6^	9.14 (3.65–22.86)
				TT	44 (66.67%)	135 (94.41%)			
7:143491644:G:A	rs1525107	7q35	EPHA1-AS1	GG	14 (21.21%)	33 (23.08%)	Dominant	5.66 × 10^−6^	0.21 (0.11–0.41)
				GA	21 (31.82%)	87 (60.84%)			
				AA	31 (46.97%)	23 (16.08%)			
11:95546774:C:T	rs2105758	11q21	Intergenic	TT	24 (36.36%)	12 (8.39%)	Recessive	2.40 × 10^−6^	6.94 (3.10–15.52)
				TC	26 (39.39%)	89 (62.24%)			
				CC	16 (24.24%)	42 (29.37%)			
12:31260391:A:G	rs622946	12p11.21	Intergenic	AA	12 (18.18%)	16 (11.19%)	Dominant	7.29 × 10^−6^	4.96 (2.46–9.99)
				AG	41 (62.12%)	50 (34.97%)			
				GG	13 (19.70%)	77 (53.85%)			
12:117767893:C:T	rs7296788	12q24.23	KSR2	CC	3 (4.55%)	28 (19.58%)	Additive	1.70 × 10^−6^	0.28 (0.16–0.47)
				CT	19 (28.79%)	75 (52.45%)	Dominant	8.48 × 10^−7^	0.20 (0.11–0.38)
				TT	44 (66.67%)	40 (27.97%)			
13:103341969:G:C	rs12857032	13q33.1	Intergenic	CC	3 (4.55%)	8 (5.59%)	Dominant	9.96 × 10^−6^	4.09 (2.19–7.65)
				CG	40 (60.61%)	38 (26.57%)			
				GG	23 (34.85%)	97 (67.83%)			

SNP, single nucleotide polymorphism; OR, odds ratio; CI, confidence interval.

The dominant model also identified significant associations for these three SNPs, along with four additional SNPs (rs16862251: OR = 0.19, *p* = 2.02 × 10^−6^, 95% CI = 0.09–0.37; rs1525107: OR = 0.21, *p* = 5.66 × 10^−6^, 95% CI = 0.11–0.41; rs622946: OR = 4.96, *p* = 7.29 × 10^−6^, 95% CI = 2.46–9.99; rs12857032: OR = 4.09, *p* = 9.96 × 10^−6^, 95% CI = 2.19–7.65). These SNPs were located in the coding regions of ANKUB1 (ankyrin repeat and ubiquitin domain containing 1) on chromosome 3q25.1, EPHA1-AS1 (EPHA1 antisense RNA 1) on chromosome 7q35, in the intergenic region between MREGP1 (melanoregulin pseudogene 1) and AC024940.5 (novel transcript, overlapping 3′UTR of FAM60A) on chromosome 12p11.21, and between SLC10A2 (solute carrier family 10 member 2) and AL162717.1 (novel transcript) on chromosome 13q33.1. The recessive model identified one significant SNP (rs2105758: OR = 6.94, *p* = 2.40 × 10^−6^, 95% CI = 3.10–15.52) in the intergenic region between AP001790.1 (novel transcript) and AP000820.2 (novel transcript) on chromosome 11q21. Manhattan plots and QQ plots depicting GWAS results for each model are presented in Supplementary Figures S1–S3.

### RSV subgroup associations

Table 3 displays the *p*-values and odds ratios for SNPs associated with the RSV G1 group. In the additive model, one SNP (rs10940848: OR = 0.15, *p* = 5.59 × 10^−6^, 95% CI = 0.07–0.34) was identified. This SNP, located in the intergenic region between AC010374.1 (piggyBac transposable element derived 3 (PGBD3) pseudogene) and RN7SKP207 (RNA, 7SK small nuclear pseudogene 207) on chromosome 5p13.3, was also significant in the dominant model (rs10940848: OR = 0.14, *p* = 4.85 × 10^−6^, 95% CI = 0.06–0.33). Furthermore, the dominant model revealed the association of another SNP, rs10127867, with the RSV G1 group (rs10127867: OR = 0.13, *p* = 7.06 × 10^−6^, 95% CI = 0.05–0.32). In the recessive model, one SNP (rs1361088: OR = 8.58, *p* = 5.21 × 10^−6^, 95% CI = 3.40–21.64), located in the coding region of KIFAP3 (kinesin-associated protein 3) on chromosome 1q24.2, was significant. Manhattan plots and QQ plots for each model are presented in Supplementary Figures S4–S6. No significant SNPs were identified in the RSV G2 group, and the corresponding Manhattan and QQ plots are shown in Supplementary Figures S7–S9.

**Table 3:** Top SNPs identified from genome-wide association analysis of RSV hospitalization in the G1 group.

SNP ID	rs ID	Location	Gene	Genotype	Patient group (*n* = 47)	Control group (*n* = 85)	Model	*p*-value	OR (95% CI)
1:170018266:A:G	rs1361088	1q24.2	KIFAP3	GG	23 (48.94%)	10 (11.76%)	Recessive	5.21 × 10^−6^	8.58 (3.40–21.64)
				GA	16 (34.04%)	49 (57.65%)			
				AA	8 (17.02%)	26 (30.59%)			
1:170049135:T:C	rs10127867	1q24.2	KIFAP3	TT	6 (12.77%)	25 (29.41%)	Dominant	7.06 × 10^−6^	0.13 (0.05–0.32)
				TC	17 (36.17%)	48 (56.47%)			
				CC	24 (51.06%)	12 (14.12%)			
5:29941574:C:T	rs10940848	5p13.3	Intergenic	TT	0 (0%)	7 (8.24%)	Additive	5.59 × 10^−6^	0.15 (0.07–0.34)
				TC	10 (21.28%)	48 (56.47%)	Dominant	4.85 × 10^−6^	0.14 (0.06–0.33)
				CC	37 (78.72%)	30 (35.29%)			

SNP, single nucleotide polymorphism; OR, odds ratio; CI, confidence interval.

### Comparison with east Asian reference frequencies

To support the observed associations, we examined whether the identified risk alleles in our case group exhibited elevated frequency patterns compared with publicly available East Asian population data. Specifically, we compared the allele frequencies of 11 candidate SNPs in our case group with those reported in the 1000 Genomes Project East Asian population. As shown in Supplementary Tables S2, S8 out of the 11 SNPs demonstrated higher frequencies in the case group: rs183825 (18.18% vs. 11.01%), rs16862251 (86.36% vs. 75.56%), rs141541148 (16.67% vs. 6.36%), rs1525107 (62.88% vs. 51.82%), rs2105758 (56.06% vs. 41.62%), rs622946 (49.24% vs. 34.51%), rs7296788 (81.06% vs. 62.63%), rs12857032 (34.85% vs. 25.76%). This frequency pattern was consistent with the observed case-control associations and may warrant further investigation.

## Discussion

In this study, we identified several genetic loci associated with an increased risk of hospitalization due to RSV infections in children in Taiwan. Our findings revealed significant associations between eight loci (rs183825, rs141541148, rs7296788, rs16862251, rs1525107, rs2105758, rs622946, and rs12857032) and the severity of RSV infection. Notably, children carrying the rs141541148 variant were nine times more likely to be hospitalized due to RSV infection compared to others, while boys with rs1361088 had an eightfold increased risk of hospitalization. We also found that individuals carrying the SNP rs16862251—a locus associated with T-cell receptor signaling—had an 80% lower rate of hospitalization due to RSV, suggesting a potential role for this pathway in modulating immune responses to RSV infection.

The genetic basis of RSV susceptibility has been widely studied, with several studies identifying genes involved in the innate immune response as key contributors. Variants in genes such as TLR4 have been associated with RSV bronchiolitis, with some studies suggesting a role for the Toll-like receptor family in recognizing pathogen-associated molecular patterns (PAMPs) during RSV infection (6). Our findings support the notion that innate immunity genes, such as those involved in T-cell signaling (e.g., ANKUB1), play crucial roles in modulating the severity of RSV disease (8) The association of rs141541148 with severe disease, for instance, is consistent with the known role of immune system genes in regulating the inflammatory response during RSV infection.

The rs16862251 polymorphism is located on the ANKUB1 gene (8). This gene codes for ankyrin repeat domains, which has a critical role in the ubiquitylation signaling pathway (USP) (8). The USP is also important for regulating T-cell receptor (TCR) signaling through the neuronal precursor cell-expressed developmentally downregulated 4 (NEDD4) protein, which increases TCR signaling to trigger the immune response (9). NEDD4 also regulates the stability of other important immune system proteins, such as c-Jun and JunB. Although the association between carrying the SNP and the risk of RSV hospitalization in children is probably related to the TCR signaling immune pathway, confirmation of this hypothesis requires further investigation. While some findings, like those related to TLR4, have been inconsistent across studies, our results contribute to a growing body of evidence suggesting that genetic variation in immune response pathways significantly impacts susceptibility to severe RSV disease.

Our study extends previous research by identifying novel genetic loci, such as rs1525107, rs7296788 and rs16862251, that have not been previously linked to RSV susceptibility. The rs16862251 polymorphism's association with a lower risk of hospitalization suggests a possible protective role mediated through T-cell receptor signaling. The finding that people who carry rs1525107 SNP (on the EPHA1-AS1 gene) have a 0.2 times increased risk of hospitalization due to RSV. EPHA1-AS1 gene, which is responsible for EphA1 signaling, a key regulator of inflammation (10). The immune responses to various infections involve the Eph receptors, which are components of receptor tyrosine kinases (10). Clinically, EphA1 deficiency can suppress pulmonary inflammation by attenuating endothelial leakage, epithelial permeability, and other inflammatory responses (11, 12). As ephrinA1/ephA2 signaling affects rhinovirus-induced innate immunity in human sinonasal epithelial cells, clinical studies have found that the expression of the ephrin A1 receptor is increased in patients with chronic rhinosinusitis (13). The rs7296788 polymorphism, located on the KSR2 gene, may contribute to increased RSV severity by enhancing MAP kinase activity, which is involved in TLR4-mediated inflammatory responses (14). Consequently, we can assume that the association between the rs7296788 SNP and the higher risk of hospitalization is a result of the immune responses to specific pathogens.

To date, three RSV vaccines have been approved for clinical use (15). Among them, the bivalent RSV prefusion F vaccine is indicated for administration during the third trimester of pregnancy to confer passive immunity to infants under 6 months of age (16). Additionally, both short-acting and long-acting monoclonal antibodies targeting RSV are available for the prevention of severe RSV infection in infants. Building on our findings and future genomic research, we aim to elucidate the genotype-specific effectiveness of RSV vaccines in early childhood, with the goal of advancing personalized immunoprophylaxis strategies.

Our study has several limitations. First, due to its retrospective and questionnaire-based design, recall bias is inevitable. Diagnoses of RSV was based on caretaker-completed questionnaires rather than medical records. Although the questionnaire was designed with logical consistency checks to reduce self-report errors, misclassification due to undocumented or asymptomatic infections cannot be ruled out. Second, recruiting participants through a social media platform presents inherent limitations, including the potential for volunteer bias and information bias. Although the page primarily targets Taiwanese parents and has a large follower base exceeding 800,000, which may help mitigate some of these biases by providing broad outreach, the sample remains non-random and self-selected. Therefore, the possibility of sampling bias affecting the generalizability of the findings cannot be excluded. Third, the analytic sample was modest (213 children after quality control), and subgroup sizes—particularly controls—were small, may limit our ability to detect additional genetic associations. Fourth, the cohort consisted predominantly of Taiwanese/East Asian children, so allele frequencies and linkage disequilibrium patterns may differ in other ancestries, may limit generalize findings to the broader population. Most candidate variants showed higher frequencies in the case group, supporting their potential role in disease susceptibility. Nonetheless, larger-scale studies with more diverse samples and clinically verified diagnoses are warranted to validate our findings.

## Conclusion

In conclusion, we have identified several gene loci associated with higher rates of hospital admissions due to RSV in children of ≥6 months of age. By studying the genetic variations that may predispose people to RSV infection, it may be possible to gain a better understanding of risk factors and prioritize vaccination for specific populations.

## Data Availability

The datasets presented in this study can be found in online repositories. The names of the repository/repositories and accession number(s) can be found in the article/Supplementary Material.
