# Supplementary material for: Genetic susceptibility associated with hospitalization due to respiratory syncytial virus in a group of Taiwanese children: a preliminary study
Source: Front Pediatr. 2025 Aug 12;13:1473448. doi: 10.3389/fped.2025.1473448 (PMC12378803; doi:10.3389/fped.2025.1473448)

**Supplementary Table**

**Supplementary Table 1.** Summary of case/control definitions for all subgroups

| Group | Case Definition | Control Definition | Sex Restriction | Region Restriction |
| --- | --- | --- | --- | --- |
| RSV G1 | Hospitalized for RSV infection | Not hospitalized for RSV | Boy | None |
| RSV G2 | Hospitalized for RSV infection | Not hospitalized for RSV | None | Taipei, New Taipei City |
| RSV All | Combination of RSV G1 and G2 (duplicates excluded) | Same as above | Combined | Combined |

**Supplementary Table 2.** Comparison of risk allele frequencies with external large-scale datasets

| SNP ID | Risk Allele | Case Frequency (%) | 1000 Genomes EAS Frequency (%) |
| --- | --- | --- | --- |
| rs183825 | T | 18.18 | 11.01 |
| rs16862251 | G | 86.36 | 75.56 |
| rs141541148 | C | 16.67 | 6.36 |
| rs1525107 | A | 62.88 | 51.82 |
| rs2105758 | T | 56.06 | 41.62 |
| rs622946 | A | 49.24 | 34.51 |
| rs7296788 | T | 81.06 | 62.63 |
| rs12857032 | C | 34.85 | 25.76 |
| rs1361088 | G | 46.97 | 49.39 |
| rs10127867 | C | 49.24 | 50.30 |
| rs10940848 | C | 63.64 | 78.48 |

**Supplementary Figure**

**Supplementary Figure 1.** Manhattan and QQ plot of GWAS results of additive model for RSV all group


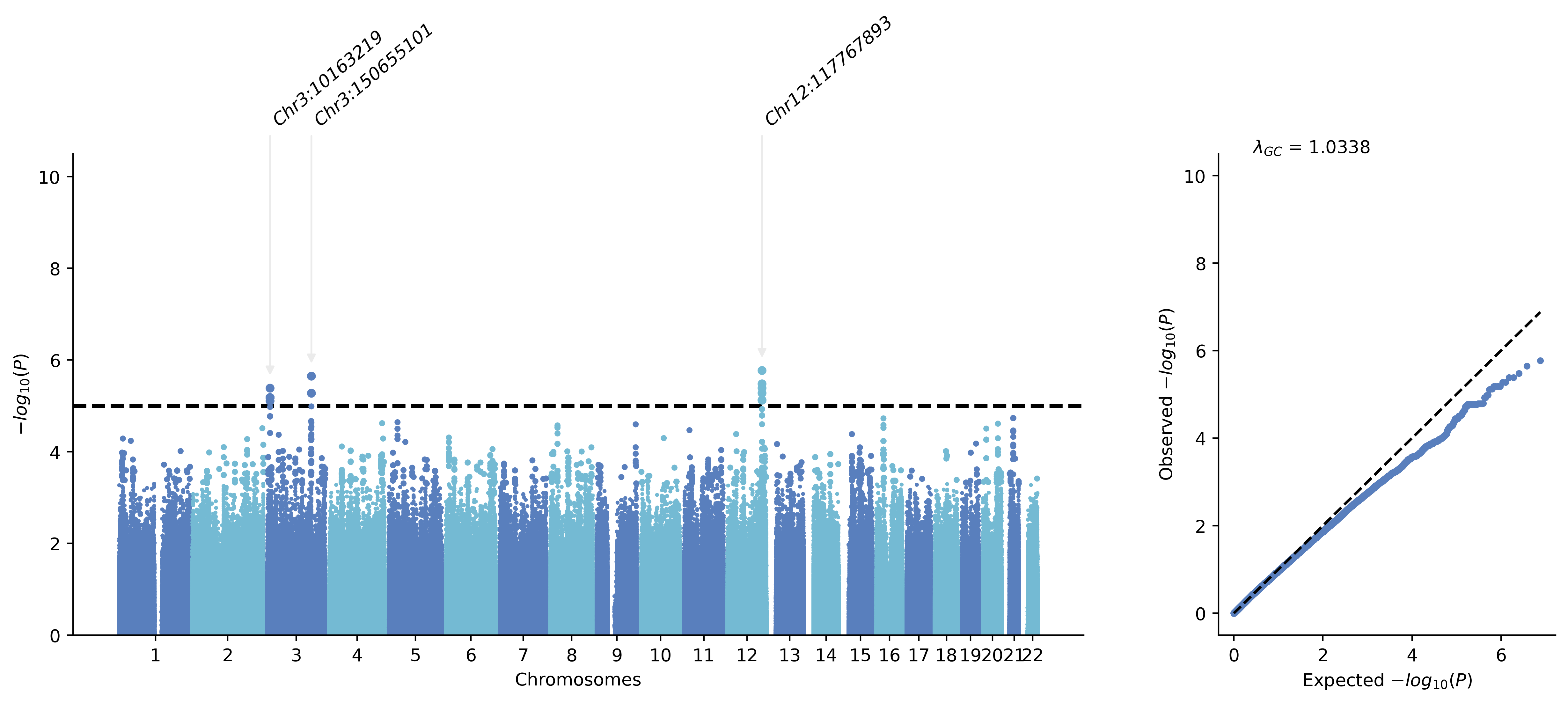


**Supplementary Figure 2.** Manhattan and QQ plot of GWAS results of dominant model for RSV all group


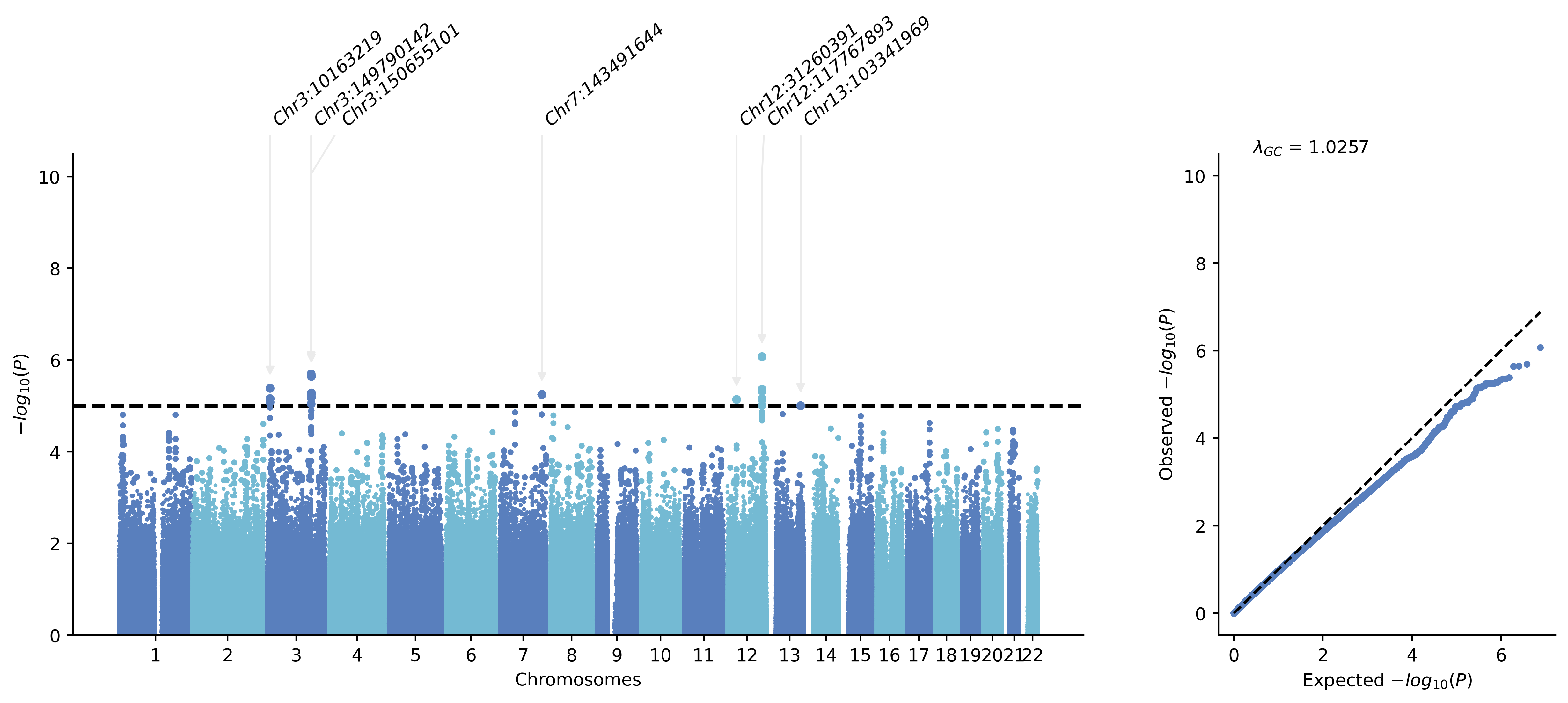


**Supplementary Figure 3.** Manhattan and QQ plot of GWAS results of recessive model for RSV all group


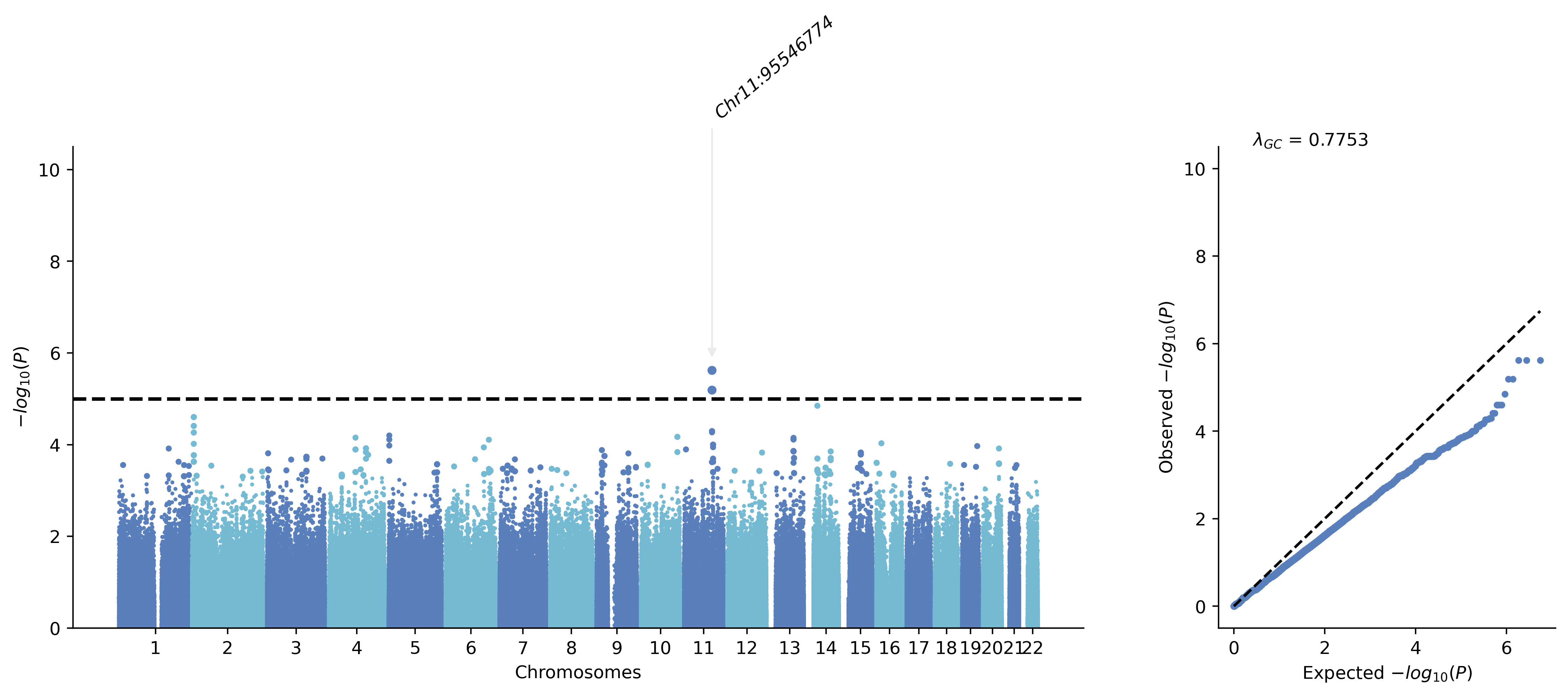


**Supplementary Figure 4.** Manhattan and QQ plot of GWAS results of additive model for RSV G1 group


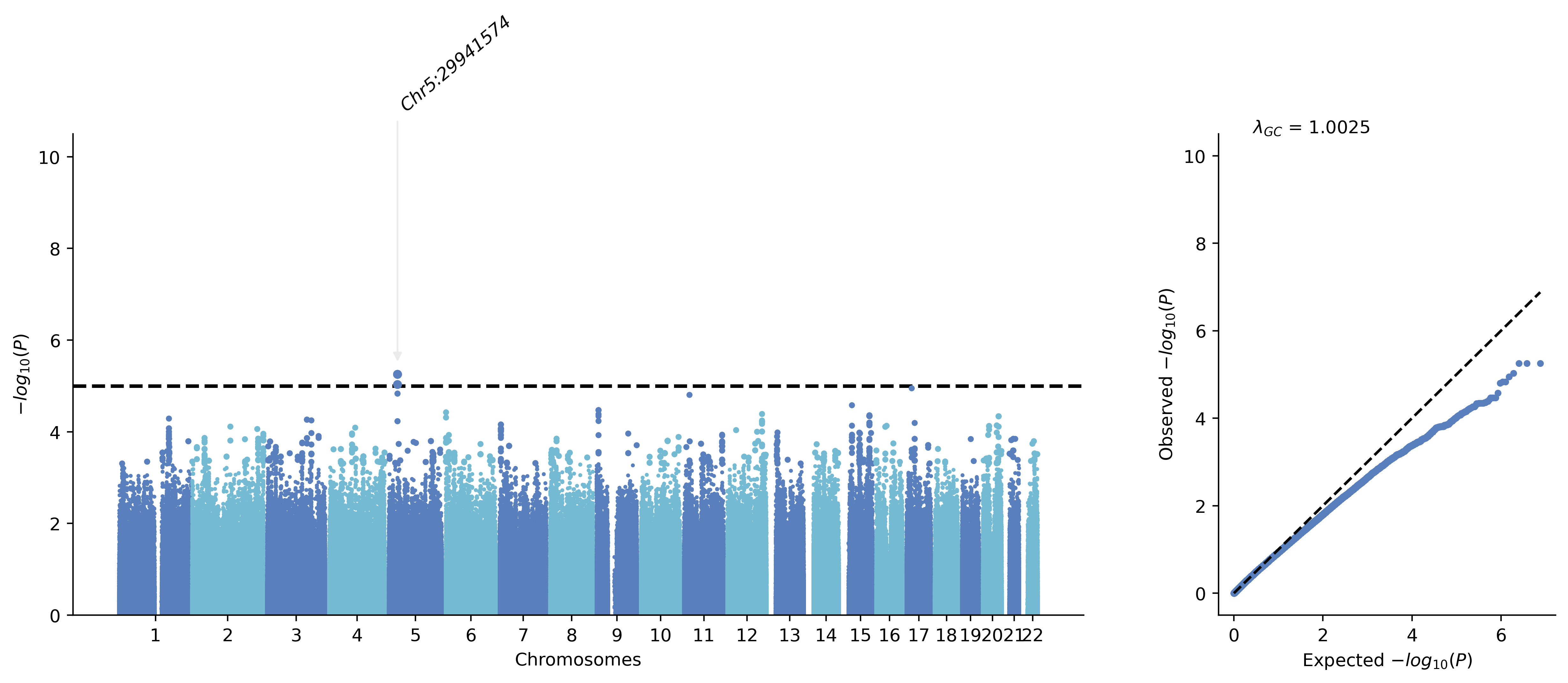


**Supplementary Figure 5.** Manhattan and QQ plot of GWAS results of dominant model for RSV G1 group


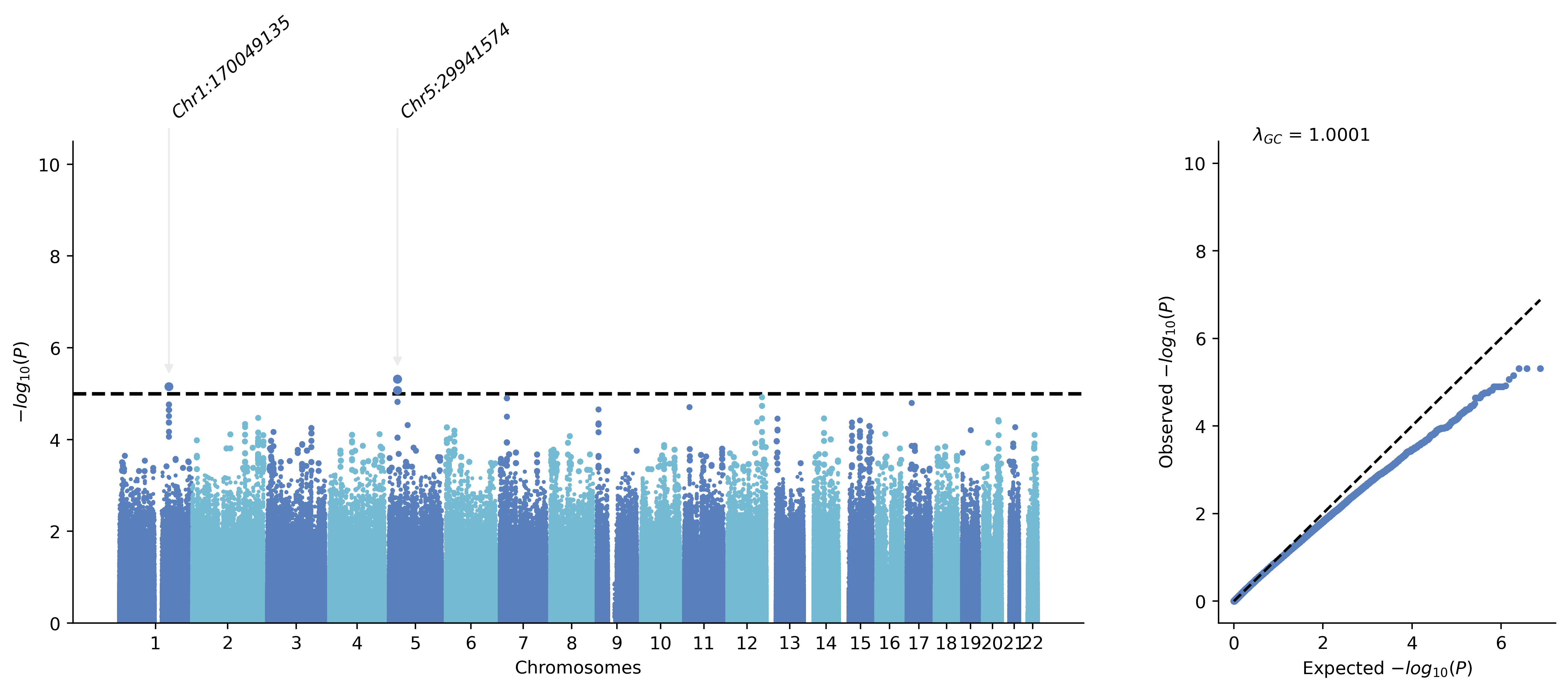


**Supplementary Figure 6.** Manhattan and QQ plot of GWAS results of recessive model for RSV G1 group


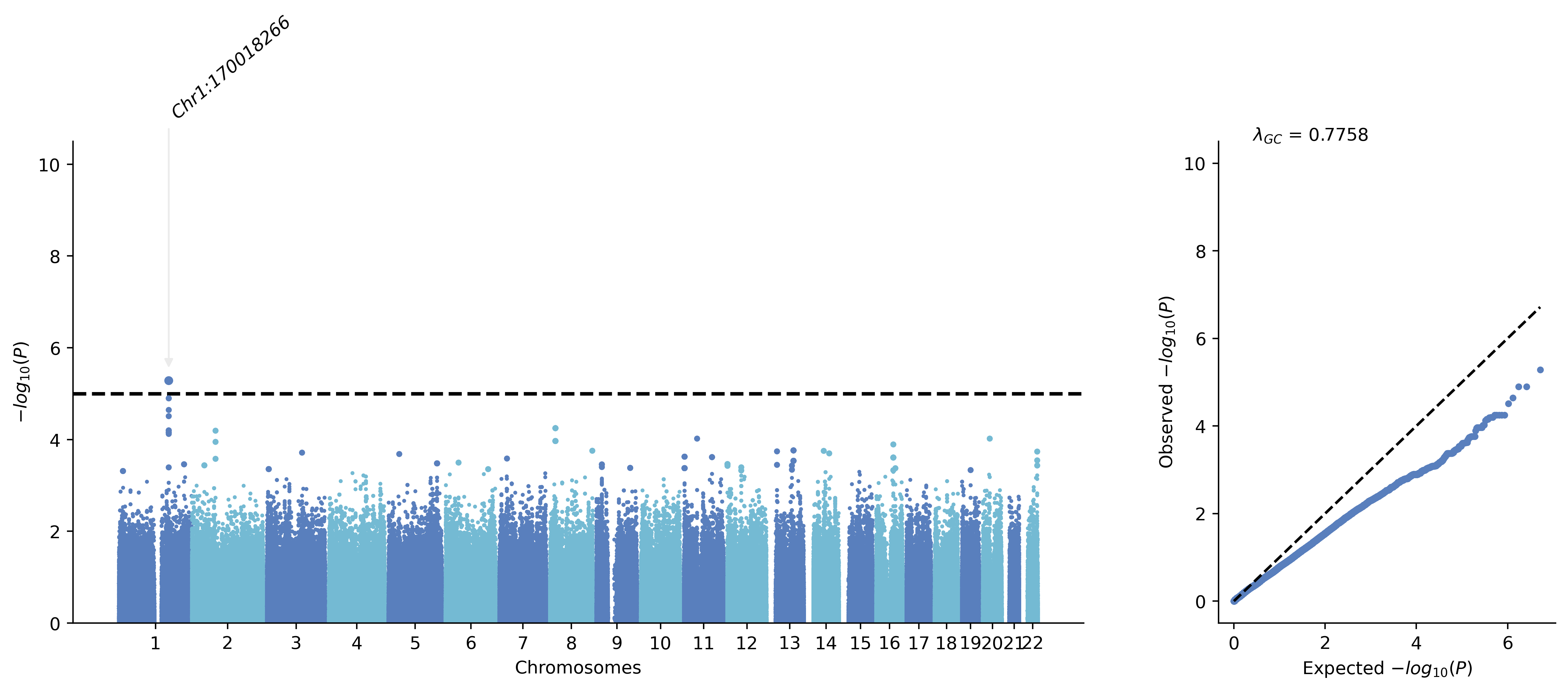


**Supplementary Figure 7.** Manhattan and QQ plot of GWAS results of additive model for RSV G2 group


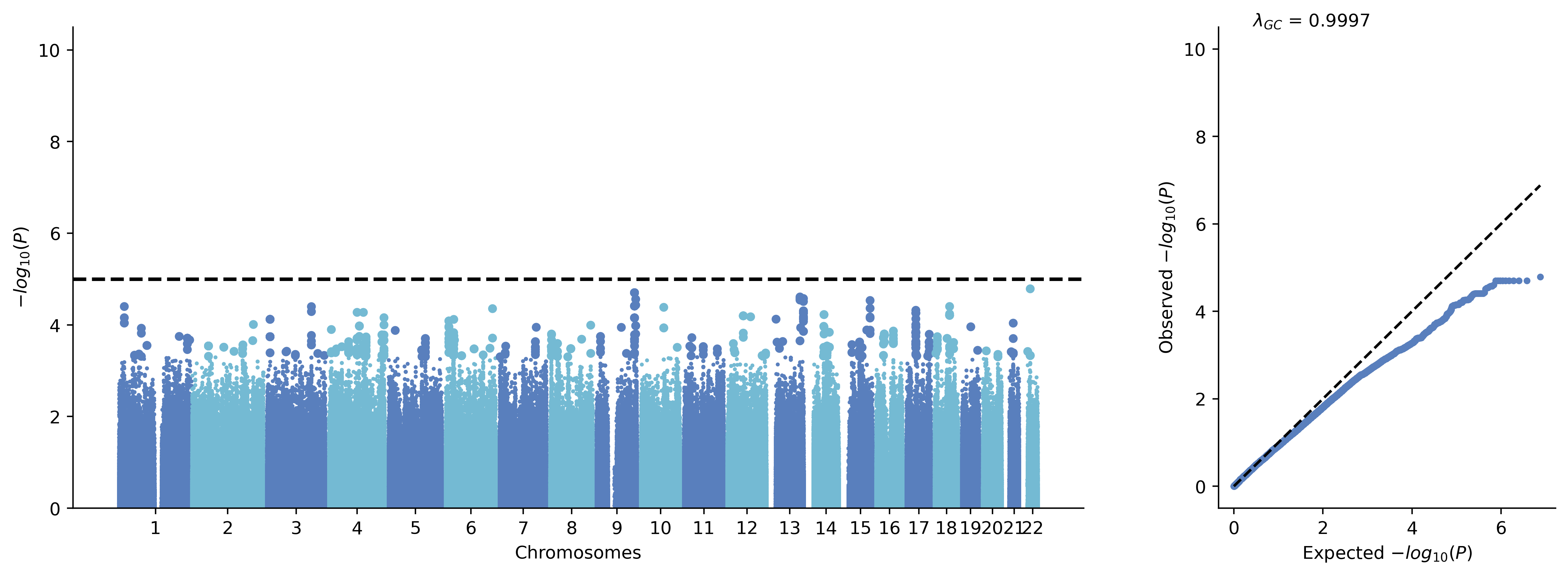


**Supplementary Figure 8.** Manhattan and QQ plot of GWAS results of dominant model for RSV G2 group


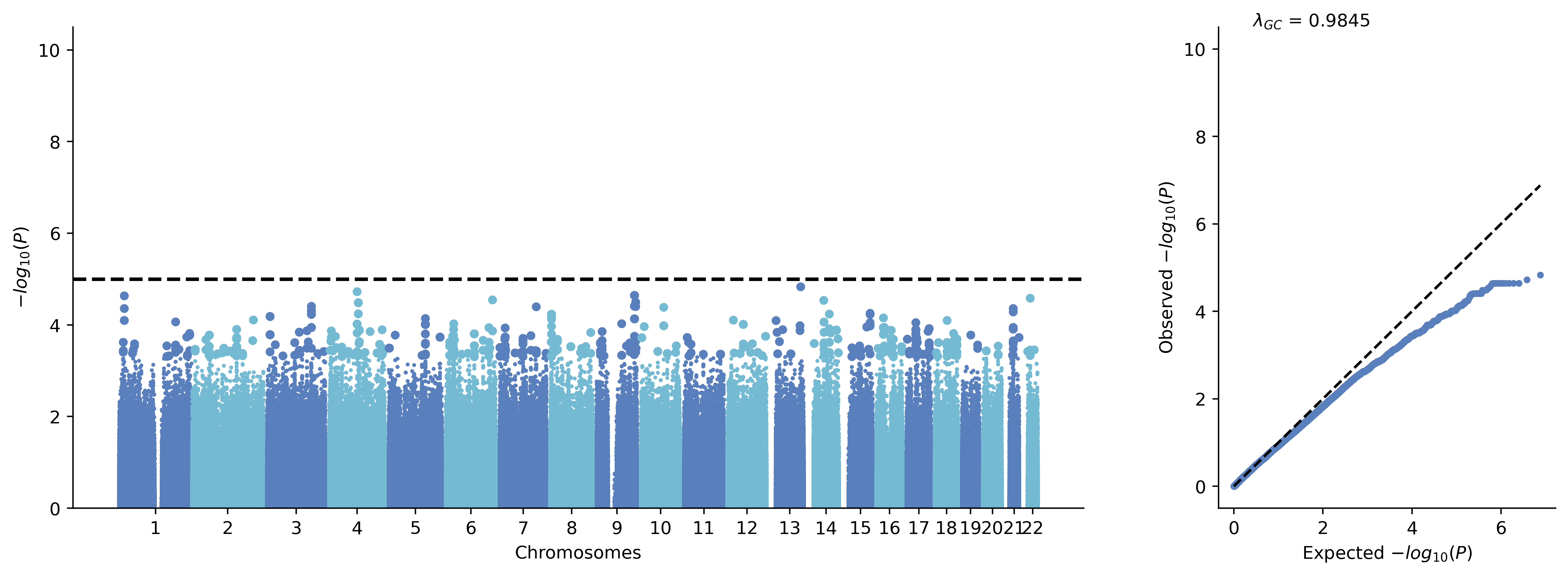


**Supplementary Figure 9.** Manhattan and QQ plot of GWAS results of recessive model for RSV G2 group


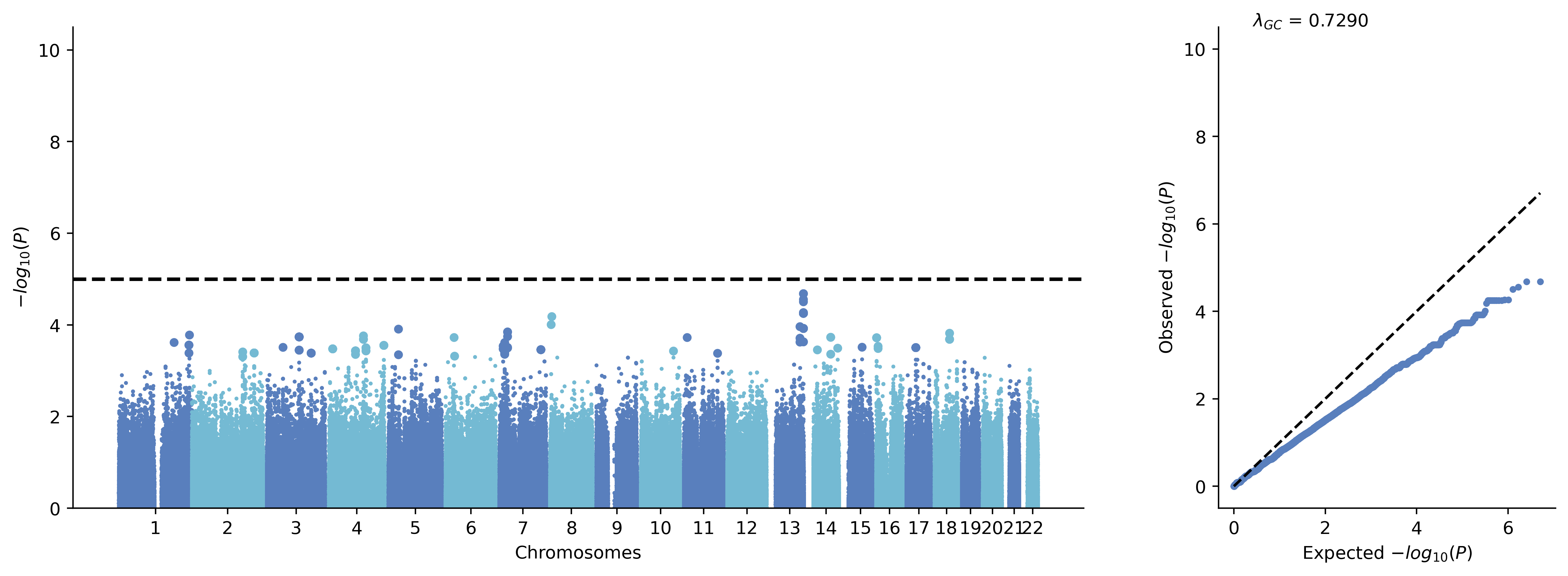

Supplement: Supplementary file 1 [file Supplementaryfile1.docx]
